# No Compass at Sea

**Published:** 1868-12

**Authors:** 


### NO COMPASS AT SEA.

It is a boon of priceless value, to have an unfaltering religious belief. One of the most affecting incidents in the history of the Divine Redeemer, occurred, when looking over the multitude, he was moved with compassion on them, "because they were as sheep having no shepherd."

That state of mind which no gold can purchase, whose value no costliest gems can express, which finds perfect repose in contemplating the present individual condition of humanity, and its future irrevocable destiny, in the expression, "The Judge of all the Earth will do right!"

Such a state of mind we say, bears with it, a sweetness of comfort, worth more than all worlds. And fortunate beyond computation is that child, whose reverence for Scripture teachings has become so incorporated with its very nature, that even in mature life the Ultima Thule as to duty and morals is, "*The Bible says it.*"

Seldom have these views had a stronger corroboration than in a meeting which we attended lately in this city. A "Shaker" was to discuss the doctrine of celibacy. The room was well-filled. The Shaker was to speak ten minutes, and any one else might reply for the same length of time. In all that assembly of men and women, we failed to discover one single countenance which indicated composure. There was an expression of anxious unrest, so general, that we were moved to pity. The women had a kind of he-look, which was grating to our feelings. There was only one female face there, to which we could turn for relief, which was found in a certain benignity of expression, which eventually cleared away that "first impression," of one of the ugliest, little, old, phizzes, which we had been lately called on to contemplate.

As to the men, there were two classes. One whose "expression" indicated that they had missed the aim of life, that they were deeply dissatisfied with their "*status*," and were seeking revengefully, for a change.

There was another set of countenances, few in comparison, but as widely different as daylight is from darkness. There was the high broad forehead, benign and intelligent, as if the owners wished all men to be happy, and felt it to be their duty to labor for that happiness; conscious of their intelligence, and of their duty to employ it in search of the true secret, of the highest human good.

In the speeches made, there was a frequent quotation of Scripture, but in such a way, as to impress us with the feeling that the quotations were made, not because of a loving and reverential confidence in Scripture authority, but from a conviction that it was authoritative, in most of those who were present. As much as to say, "you see I am on the side of the Bible, do not be afraid of me, as of an infidel."

Be assured reader, that no scheme of human amelioration ever can succeed, where the Bible is not received "IN THE LOVE OF IT." Hence, the miserable failures of Ann Lee, of Fourier, of Brisbane, of Owen, and the thousand and one modifications of the Agrarian of ancient times, of the Arcadian and the Philansters of the present.

To make all men happy, we must first make them unselfish, in obedience to the Bible precept, "Thou shalt love thy neighbor as thyself." But when that is done, the world becomes truly religious, and nothing more is needed.

As a means then, of making earth a paradise, where love, and intelligence and plenty, shall universally prevail, leaving no room for dissatisfaction, disquietude, for wasting anxieties and corroding cares, nor for want and famine and *disease*, we earnestly commend one item of early education—

*Implant into the very nature of your children, from earliest infancy, an affectionate and implicit belief in* ALL *Bible Teachings.*

